# Acute Traumatic Spondyloptosis: A Case Report

**DOI:** 10.7759/cureus.36457

**Published:** 2023-03-21

**Authors:** Johann Braithwaite, Joshua Gruber, Jordan Fakhoury, Gus Katsigiorigis, Kanwarpaul Grewal

**Affiliations:** 1 Department of Orthopaedic Surgery, Orthopedic Surgery Residency Program, Donald and Barbara Zucker School of Medicine at Hofstra/Northwell, Huntington, USA; 2 Department of Orthopaedic Surgery, Nova Southeastern University Dr. Kiran C. Patel College of Osteopathic Medicine, Fort Lauderdale, USA

**Keywords:** lumbosacral dissociation, surgical intervention, asia e, fusion, trauma, spine, spondylolisthesis, spondyloptosis, traumatic, acute

## Abstract

Acute traumatic spondyloptosis (ATS) is a rare condition in the orthopedic literature, with few cases reported. We present a case of ATS in a 35-year-old Hispanic male with multilevel injury, without neurological deficits at the time of injury. The patient was treated in a two-stage method consisting of combined anterior and posterior spinal decompression and fusion. At the six-month follow-up, the patient had no motor/sensory deficits, he remained stable during the one-year period. Conclusion: ATS is rarely seen in patients without neurological deficits on presentation. Although surgical intervention presents significant risks of iatrogenic neurologic compromise, surgical fixation is warranted.

## Introduction

Acute traumatic spondyloptosis (ATS) is a rare injury resulting from high-energy impact creating an unstable injury requiring surgical reconstruction and stabilization [1-3]. Furthermore, severe translational spinal injuries and their resulting deformities disrupt the vertebral column and the adjacent structures, leading to neurological deficits [4]. Although severe neurological impairment is expected with traumatic spondyloptosis, there have been cases of neurologically intact patients [3,5]. We report a unique case of a neurologically intact patient following ATS at the L5-S1 level after a reported fall. The patient underwent staged reconstructive surgery with lumbar decompression L1-S1 and posterior spinal fusion T11-S1, followed by lateral interbody fusion L3-4, and L5-S1 anterior lateral interbody fusion. We describe the clinical presentation and surgical management of this unique case.

## Case presentation

**Figure 1:**
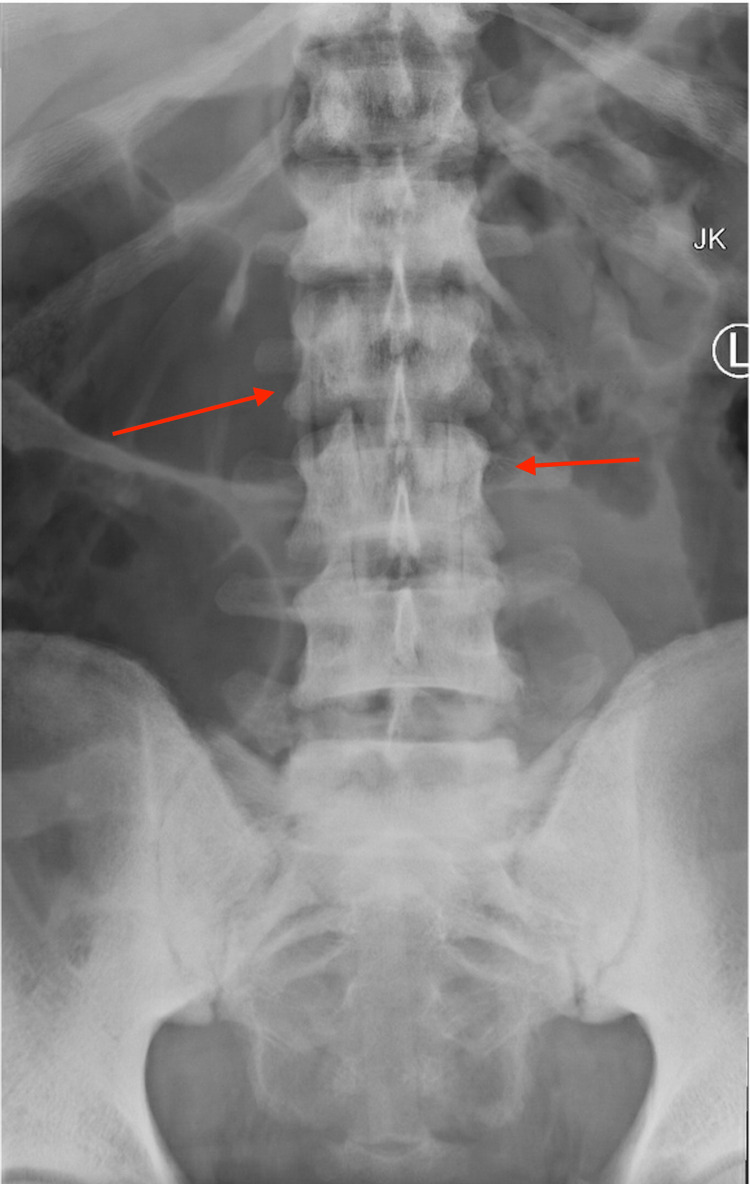
Anterior-posterior X-ray of the lumbar spine; initial injury X-ray in the emergency room.

**Figure 2:**
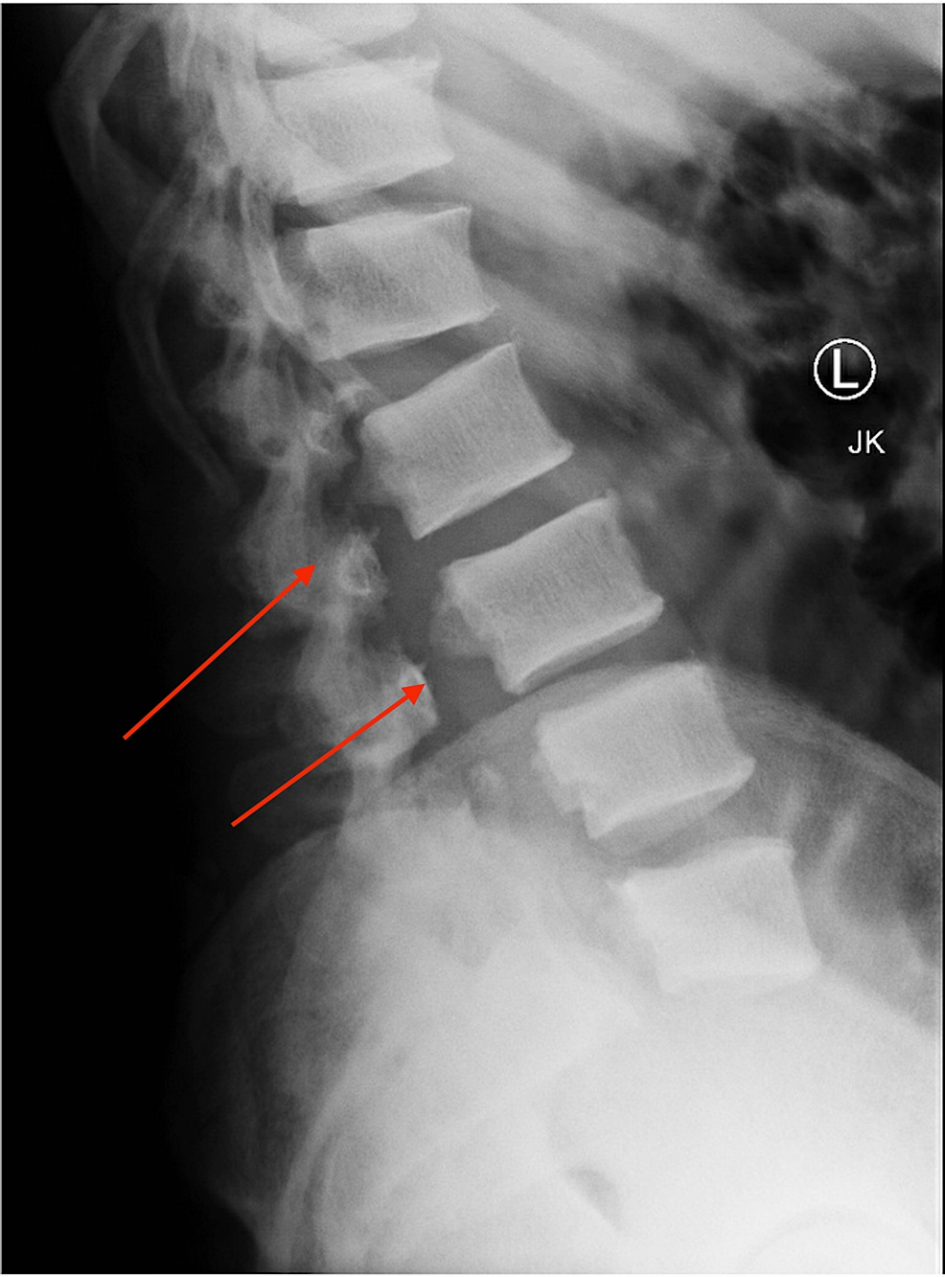
Lateral X-ray of the lumbar spine; initial injury X-ray demonstrating greater than 100% anterolisthesis L5-S1 with bilateral pedicle fractures L2-5.

**Figure 3:**
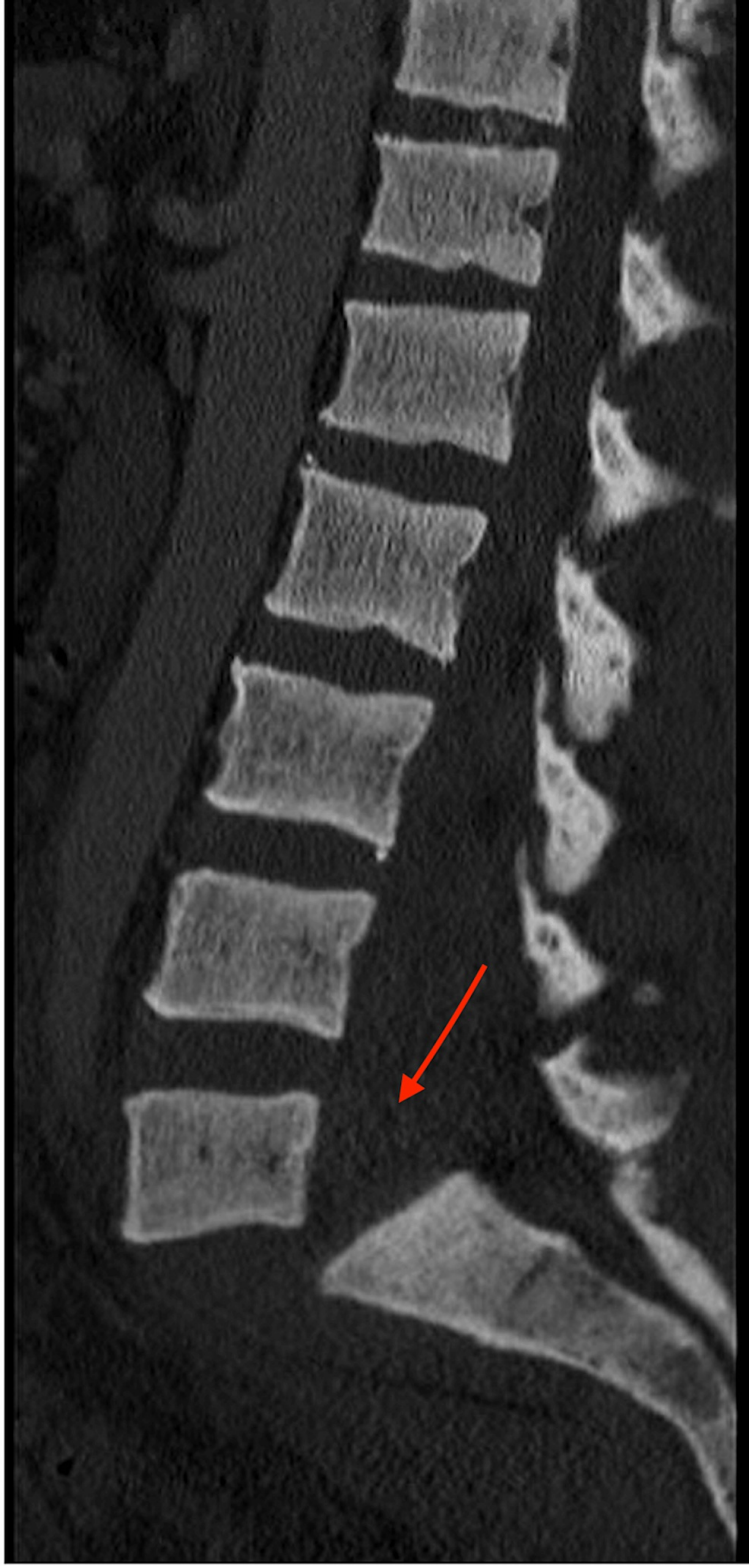
CT lumbar spine sagittal sequence at initial presentation demonstrating bilateral pedicle fractures of L2-5 and spondyloptosis of L5-S1, causing “en bloc” lumbosacral dissociation.

**Figure 4:**
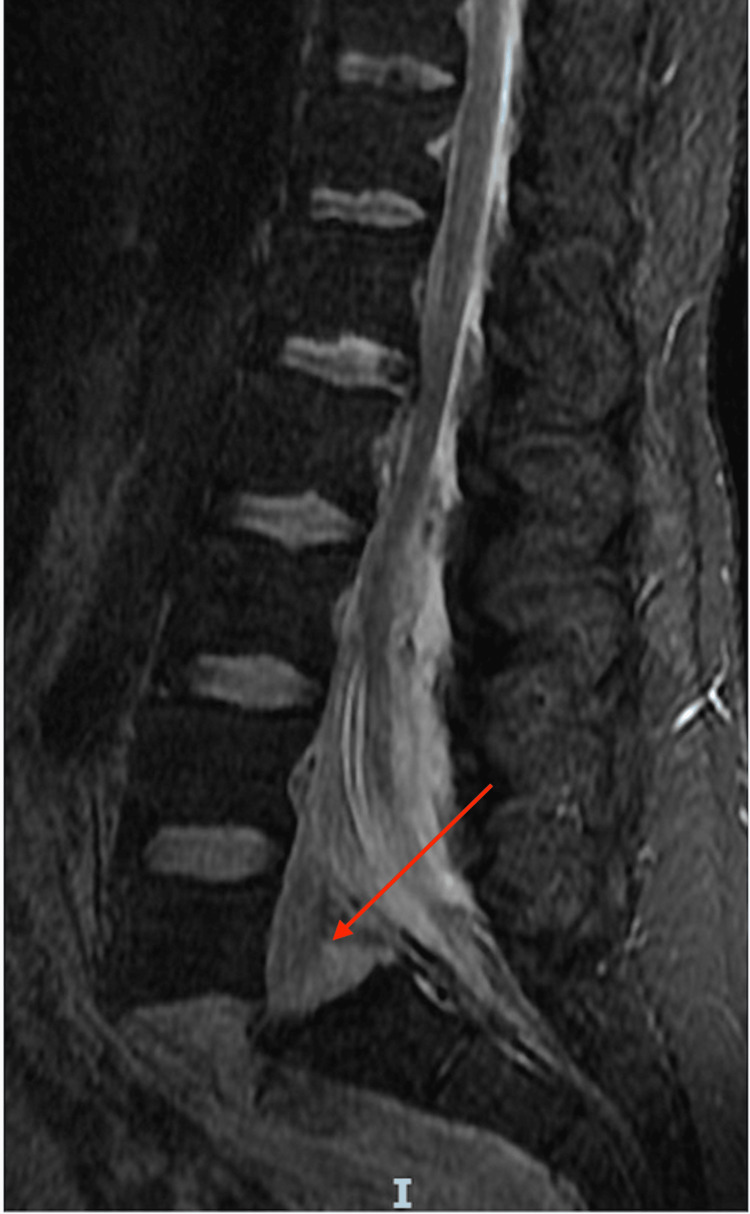
T2 MRI lumbar spine sagittal sequence demonstrated disruption of the anterior and posterior longitudinal ligaments, complete disruption of the L5-S1 intervertebral disc, and mass effect on the lumbosacral nerve roots (cauda equina).

A 35-year-old Hispanic male with no previous medical history or surgeries presented to our emergency department complaining of intractable lower back pain and inability to ambulate upon awakening. The night before, he fell down a flight of steps and reportedly ambulated back to bed. On presentation, he was neurologically intact (L2-S1 5/5 motor strength, no sensory deficits, no upper motor neuron signs, rectal tone/perirectal sensation intact), and was graded E on ASIA E (American Spinal Injury Association Impairment Scale), a standardized neurological scale used to assess sensory and motor levels impacted by spinal cord injury, in which grade E indicates that the patient's sensory and motor functions are normal and grade A indicates complete impairment below the level of injury. Lumbar spine X-ray showed ATS at L5-S1 and bilateral pedicle fractures of L2-5 (Figures 1-2). Lumbar spine CT confirmed bilateral L2-5 pedicle fractures and L5-S1 spondyloptosis, causing lumbosacral dissociation with vertebral alignment remaining intact L2-L5 (Figure 3). CT also revealed epidural/foraminal hemorrhage at L2-5, bilateral L5 transverse process fractures, and retroperitoneal hemorrhage. MRI additionally demonstrated anterior and posterior longitudinal ligament disruption, complete L5-S1 intervertebral disc disruption, and mass effect on the lumbosacral nerve roots (cauda equina) secondary to epidural hemorrhage (Figure 4).

The orthopedic spine specialist was consulted and discussed surgical fixation risks/benefits with the patient, including loss of baseline function/paralysis due to the procedure. A two-staged procedure was planned, with initial posterior spinal instrumentation and subsequent anterior augmentation via anterior/lateral interbody fusion for this unstable fracture. 

The patient was medically optimized and taken to the operating room on the second day of hospitalization. The first procedure involved spinal decompression and posterior-lateral thoracic/lumbar spinal fusion T11-S1 with a four-rod technique. After neuromonitoring leads were placed and motor functions were tested and recorded to obtain baseline levels, a closed reduction was attempted to realign the spine through non-invasive longitudinal traction and flexion of the lumbar spine. Fluoroscopic imaging demonstrated failure of the reduction attempt. The patient was then placed in a prone and flexed position on a Wilson frame (Mizuho OSI, Union City, CA) (a table used to keep a patient in the flexed position often used for lumbar procedures), and closed reduction was again unsuccessful with axial traction on the legs. The posterior spine was exposed from the T11-sacrum, using a standard posterior approach. An iliac crest osteotomy was performed to harvest bone graft and to place iliac bolts. Next, screws were placed in the iliac crest and S1 bilaterally. Attention was then focused on L1, where pedicle screws were placed bilaterally. Instrumentation to fixate L2-5 was deferred at this time due to fracture-dislocation of the pedicles. Pedicle screws were placed at T11-T12. Instrumentation at T10 was deferred from the initial preoperative plan due to osteosclerotic bone found intraoperatively, thus creating a risk of inadequate fixation at lower levels. Following the prior steps, neuromonitoring was reassessed to be at baseline.

Lumbar decompression was conducted with complete L1-S1 laminectomy using the Smith-Peterson technique (an osteotomy technique used for spinal deformity through mobile spinal segments) followed by complete bilateral L4-L5 facetectomy. A large epidural hematoma was evacuated, which demonstrated injury to epidural vessels. Next, under direct visualization of the L4 nerve roots, bilateral screws were placed in the vertebral bodies to act as artificial pedicles. Pre-contoured rods were then placed bilaterally from the level of T11 to the pelvis and the displaced columns of the spine were reduced to the rods. Post reduction, when the spine was sufficiently realigned, motor/sensory readings were lost on neuromonitoring. Fixation was loosened and reduction was relaxed in an attempt to correct the neurologic deficits; however, the spine fell into its natural resting position and did not displace back to misalignment. The epidural hematoma was suspected to be causing compression and neurological deficits prior to its evacuation.

Following fixation, more stability of the lower lumbar spine was deemed necessary and bilateral L5 screws were placed into the vertebral body of L5 to create artificial pedicles. W connectors (an attachment used to apply two rods to one pedicle screw allowing four rods to be placed into the vertebrae thus referred a quad rod construct) were placed proximally and distally to accommodate a quad rod construct. Accessory rods were placed bilaterally, and the spine was reduced to this construct. Post-reduction neuromonitoring showed restored baseline motor/sensory function except for the tibialis anterior muscles bilaterally. Set screws were then tightened and final X-rays were obtained. The posterior incision was closed, and drains were left in place. This first operation presented only the initial stabilization; further surgery remained necessary for additional anterior stabilization of the spine.

The second stabilization stage consisted of an anterior L5-S1 interbody fusion on hospital day ten. The patient was placed supine on a flat-top Jackson (Mizuho OSI, Union City, CA) (a table often used in orthopedic spinal surgeries), and the procedure was conducted via an anterior retroperitoneal approach. A large portion of the L5-S1 disc was found to be sheared out anteriorly. The disc was removed and an implant (NuVasive, San Diego, CA) with 10 degrees of lordosis was prepared with a bone graft (NuVasive, San Diego, CA). The implant was placed under X-ray guidance with two screws into S1 and one screw into L5. Intraoperative assessment revealed an unsafe anterior approach to the L4-5 disc due to a hematoma and scar tissue obscuring the visibility of nearby vessels.

The wound was closed, and the patient was placed in a lateral recumbent position. The lateral spine was approached in the direct lateral approach, and fluoroscopy confirmed the L3-L4 disc space. A freehand stimulation check for nerve function was conducted to ensure no nerves were in the field; the retractors (NuVasive, San Diego, CA) were placed, and visualization was achieved. A discectomy was performed at the L3-4 disc space, and an interbody implant (NuVasive, San Diego, CA) was placed under fluoroscopic guidance. Bone morphogenetic proteins (BMP) and a bone graft (NuVasive, San Diego, CA) were used in the cage interbody implant, and then a four-hole plate was placed in the lateral aspect of the spine. Next, a separate fascial blunt incision was made down to L2-L3. The preoperative plan included an L2-3 interbody fusion; however, poor visualization and positioning of the ribs inhibited adequate access to L2-3. Final images were taken, wounds were irrigated, and standard closing was performed. The patient was extubated and recovered with no obvious complications. 

**Figure 5:**
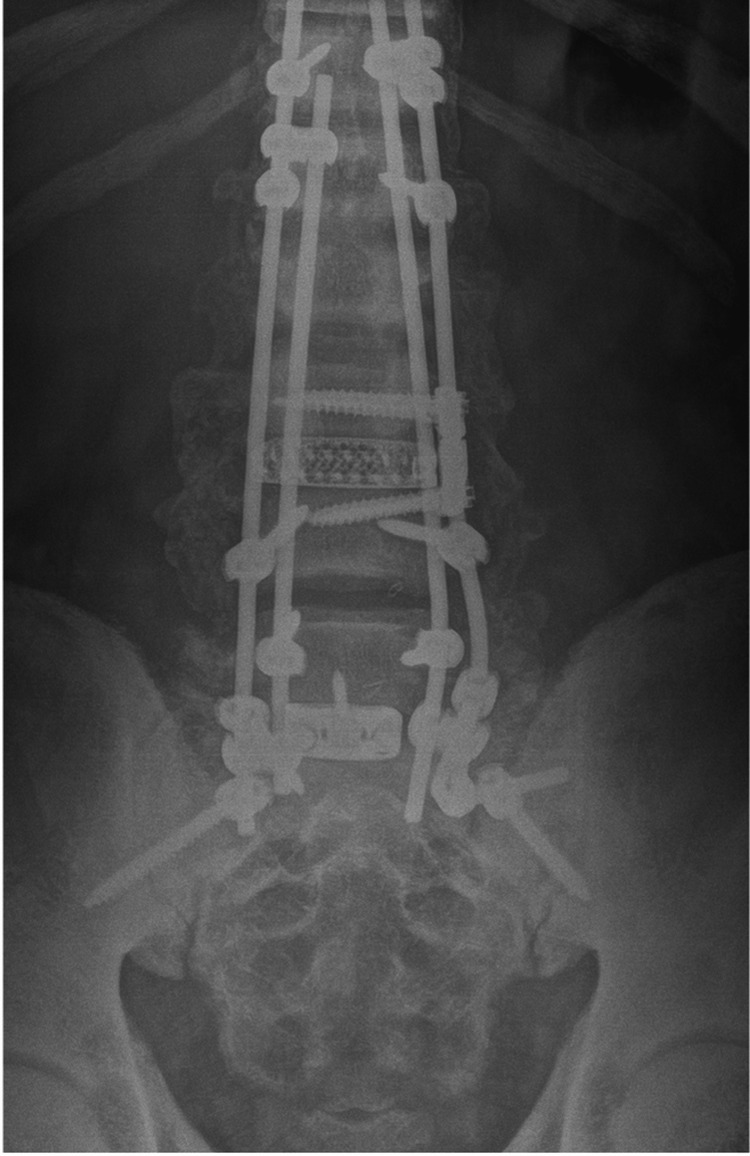
One-year follow-up anterior-posterior X-ray lumbar spine demonstrating evidence of fusion, good coronal alignment, and well-fixed orthopedic hardware.

**Figure 6:**
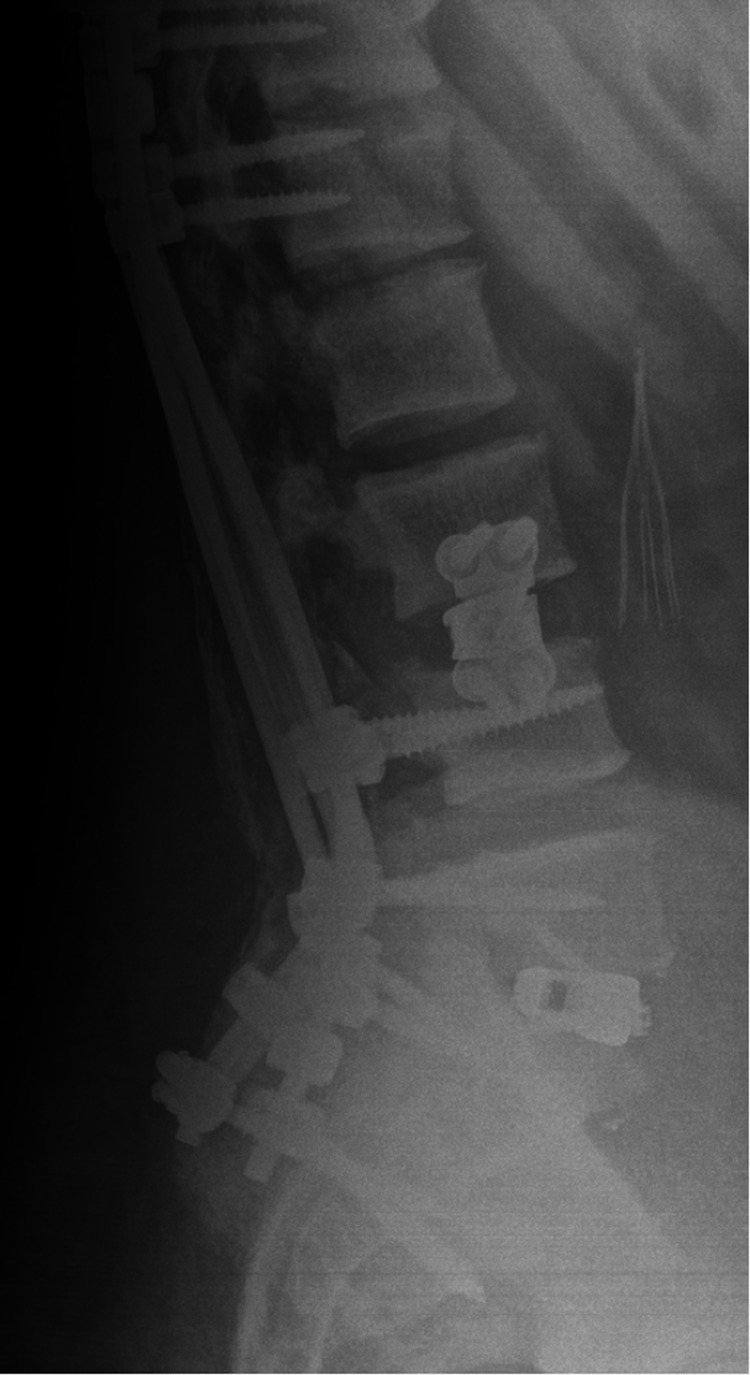
One-year follow-up lateral X-ray lumbar spine demonstrating evidence of fusion, good sagittal alignment, and well-fixed orthopedic hardware.

**Figure 7:**
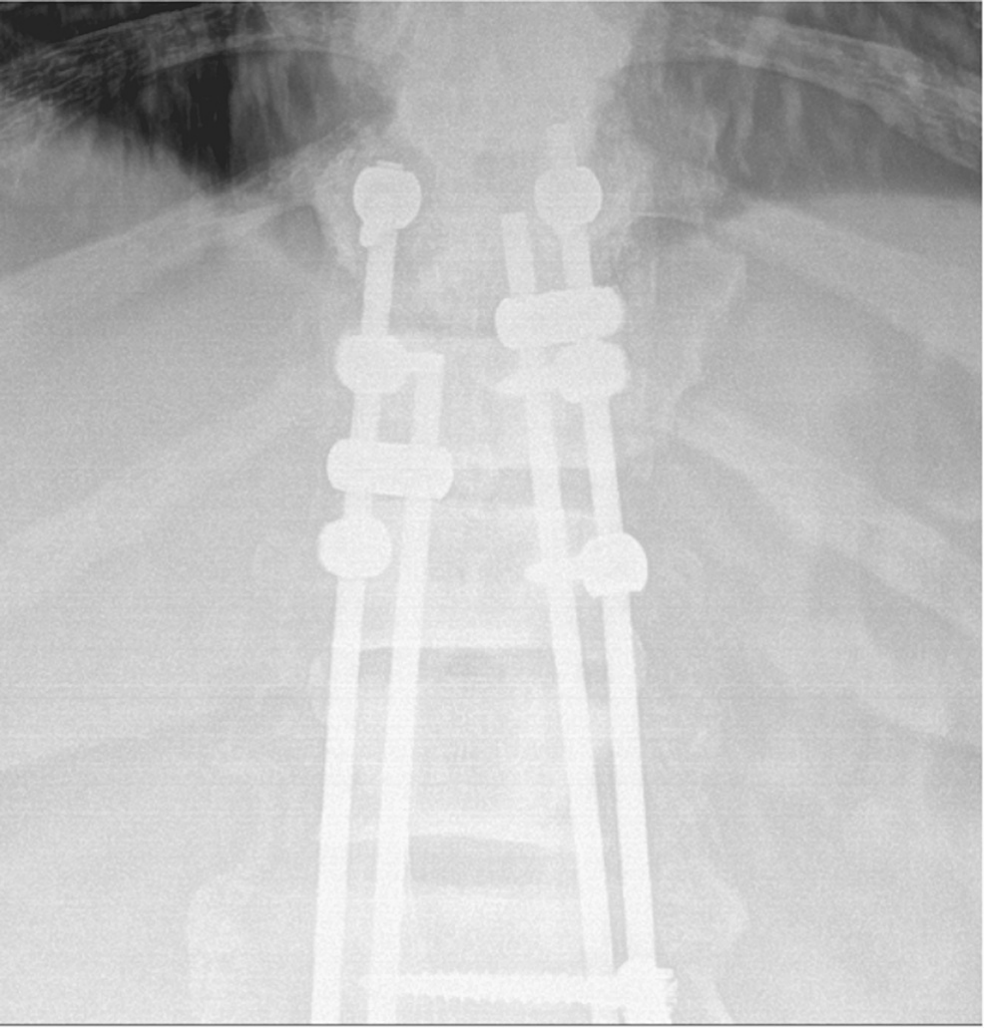
One-year follow-up AP X-ray of the thoracic spine demonstrating proximal extent of orthopedic hardware with evidence of fusion and good coronal alignment.

**Figure 8:**
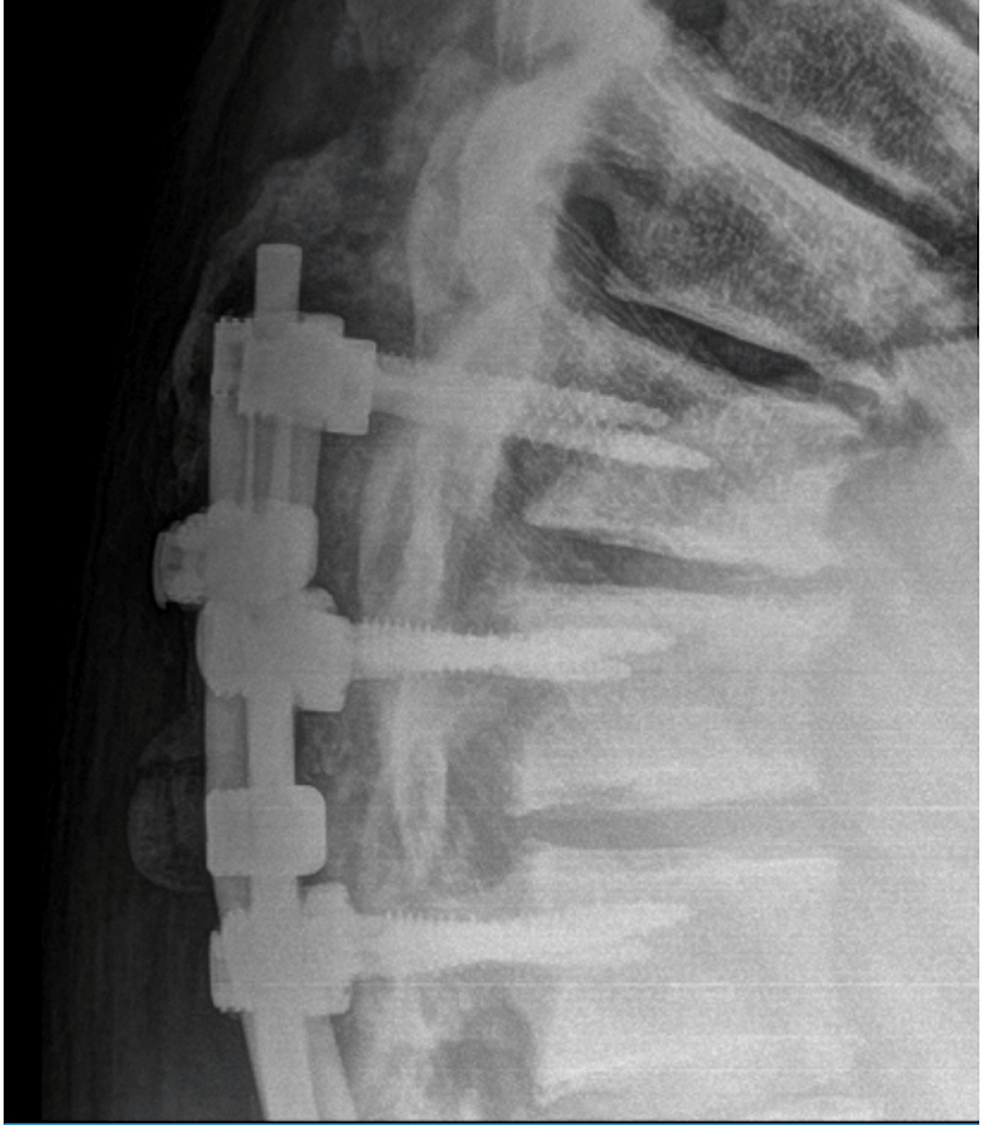
One-year follow-up lateral X-ray thoracic spine demonstrating proximal extent of orthopedic hardware with evidence of fusion and good sagittal alignment.

Postoperatively, the patient was placed in bilateral ankle-foot orthoses for bilateral tibialis anterior and extensor hallucis longus muscle weakness. The postoperative period was complicated by bilateral lower extremity deep vein thromboses. The patient was medically stable and discharged to rehab on hospital day fourteen. After one month at rehab, the patient was discharged home with home care. At one year follow-up, anterior-posterior/lateral lumbar films demonstrated acceptable alignment of the lumbosacral spine with evidence of fusion (Figures 5-8). The patient regained motor function bilaterally, no longer requiring orthoses since his six-month follow-up.

## Discussion

Spondyloptosis is the rarest and most severe form of spondylolisthesis, with only 5% of injuries involving the lumbar spine [6]. Our patient’s lack of neurological deficits upon presentation made this case even more peculiar. Similar injuries of ATS involving the thoracic spine show severe neurologic deficits in 80% of cases [7]. ATS requires reconstructive surgery to restore stability and alleviate spine and spinal canal deformities. Due to the complex nature of the injury and associated complications, treatment generally requires a multi-staged procedure [8]. This case was no exception; it was determined that a two-stage procedure was necessary to provide stabilization to the lumbar spine and prevent neurologic impairment.

The two-stage reconstruction used in our case involved decompression and T11-S1 spinal fusion followed by anterior L5-S1 interbody fusion. The first stage of the procedure involved a posterior approach due to its lower rates of mortality and procedure-related complications when compared to an anterior lumbar approach [9]. L5-S1 fusion has been used in cases of L5-S1 ATS and has proven to be effective in restoring stabilization and neurologic function. While this procedure has proven successful, it carries the risk of iatrogenic injury to the spinal cord and stretching of the cauda equina, thus resulting in neurologic dysfunction [10].

Other surgical options, including the Gaines procedure (a surgical procedure for spondyloptosis which requires a separate anterior and posterior approach to the lumbosacral junction) and its variations, have shown success in previous cases with neurologic impairment upon presentation. While effective, they carry a high risk for severe neurologic impairment [10,11]. Posterior instrumentation using a rod and screw system has been suggested to be a useful method for reduction, stabilization, and decompression of L5-S1 spondylolisthesis [12]. In the second stage of our procedure, we performed anterior interbody fusion. Previous literature has demonstrated that in cases of spondyloptosis with intervertebral disc lesions, posterior or anterior interbody fusion should be performed to achieve stabilization [13].

Surgery is recommended to correct deformity, optimize recovery for neurologically impaired patients, and prevent further neurologic injury [13]. Our goal was to stabilize the spine to prevent neurologic injury. However, this patient suffered iatrogenic motor deficits at the bilateral tibialis anterior and extensor hallucis longus muscles secondary to spinal reduction. After successful posterior and anterior lumbosacral stabilization, the patient regained all motor function at six months follow-up and remained stable at one- and two-year follow-ups. 

## Conclusions

Acute traumatic spondyloptosis is a rare injury that is unlikely to present without signs of neurological impairment. Reconstructive surgery is used to stabilize the spine, thus preventing spinal cord compression in the spinal canal. These procedures present risks of iatrogenic neurologic injury, however, they are necessary to address immense instability caused by spondyloptosis.
